# Supplementary material for: Drugs That Mimic Hypoxia Selectively Target EBV-Positive Gastric Cancer Cells
Source: Cancers (Basel). 2023 Mar 19;15(6):1846. doi: 10.3390/cancers15061846 (PMC10046841; doi:10.3390/cancers15061846)
Supplement: Supplementary file 1 [file cancers-15-01846-s001.zip › cancers-2235226-SUPPLEMENTARY.pdf]

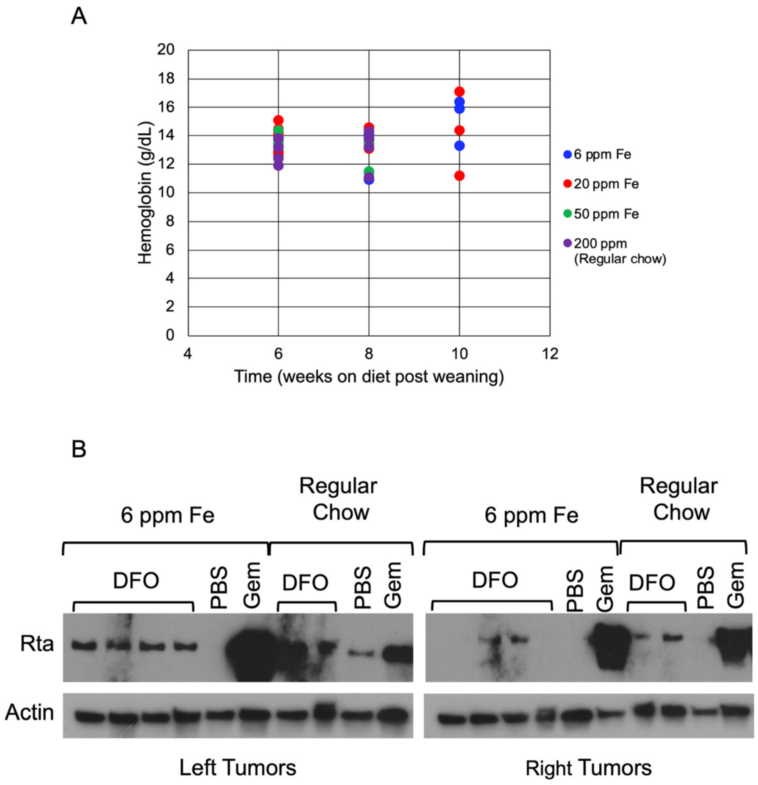

Figure S1. A low-iron diet for 10 weeks post weaning fails to induce either anemia or to enhance EBV reactivation by DFO. From weaning, groups of 5–12 mice each were fed diets containing 20-ppm Fe, 50-ppm Fe, or regular chow (200-ppm Fe). After 4 weeks, the mice were injected with AGS-Akata cells into both flanks. At this time, some of the mice receiving the 20-ppm Fe diet were switched to a 6-ppm Fe diet; the rest continued their post-weaning diet. (**A**) Maxillary vein bleeds were performed at 6, 8 and 10 weeks post weaning and hemoglobin values determined (**B**). At 10 weeks, mice were injected i.p. with DFO, gemcitabine, or PBS and processed for immunoblot analysis as described in the legend to Figure 7.
